# Trichostatin A Alleviates Renal Interstitial Fibrosis Through Modulation of the M2 Macrophage Subpopulation

**DOI:** 10.3390/ijms21175966

**Published:** 2020-08-19

**Authors:** Wei-Cheng Tseng, Ming-Tsun Tsai, Nien-Jung Chen, Der-Cherng Tarng

**Affiliations:** 1 Division of Nephrology, Department of Medicine, Taipei Veterans General Hospital, Taipei 11217, Taiwan; wctseng@gmail.com (W.-C.T.); mingtsun74@gmail.com (M.-T.T.); 2 Faculty of Medicine, School of Medicine, National Yang-Ming University, Taipei 11221, Taiwan; 3 Institute of Clinical Medicine, School of Medicine, National Yang-Ming University, Taipei 11221, Taiwan; 4 Center for Intelligent Drug Systems and Smart Bio-devices (IDS2B), National Chiao-Tung University, Hsinchu 30010, Taiwan; 5 Institute of Microbiology and Immunology, School of Life Sciences, National Yang-Ming University, Taipei 11221, Taiwan; njchen@ym.edu.tw; 6 Department and Institute of Physiology, School of Medicine, National Yang-Ming University, Taipei 11221, Taiwan; 7 Department of Biological Science and Technology, College of Biological Science and Technology, National Chiao-Tung University, Hsinchu 30010, Taiwan

**Keywords:** macrophage subpopulation, renal fibrosis, trichostatin A

## Abstract

Mounting evidence indicates that an increase in histone deacetylation contributes to renal fibrosis. Although inhibition of histone deacetylase (HDAC) can reduce the extent of fibrosis, whether HDAC inhibitors exert the antifibrotic effect through modulating the phenotypes of macrophages, the key regulator of renal fibrosis, remains unknown. Moreover, the functional roles of the M2 macrophage subpopulation in fibrotic kidney diseases remain incompletely understood. Herein, we investigated the role of HDAC inhibitors on renal fibrogenesis and macrophage plasticity. We found that HDAC inhibition by trichostatin A (TSA) reduced the accumulation of interstitial macrophages, suppressed the activation of myofibroblasts and attenuated the extent of fibrosis in obstructive nephropathy. Moreover, TSA inhibited M1 macrophages and augmented M2 macrophage infiltration in fibrotic kidney tissue. Interestingly, TSA preferentially upregulated M2c macrophages and suppressed M2a macrophages in the obstructed kidneys, which was correlated with a reduction of interstitial fibrosis. TSA also repressed the expression of proinflammatory and profibrotic molecules in cultured M2a macrophages and inhibited the activation of renal myofibroblasts. In conclusion, our study was the first to show that HDAC inhibition by TSA alleviates renal fibrosis in obstructed kidneys through facilitating an M1 to M2c macrophage transition.

## 1. Introduction

Chronic kidney disease (CKD) is an emerging global public health issue with a prevalence rate of 10% to 12% worldwide [1]. Regardless of the etiologies of renal diseases, unresolved renal insult engages an excessive deposition of extracellular matrix in the tubulointerstitium, thereby bringing about end-stage renal disease [2]. Tubulointerstitial fibrosis is the final common pathway of all kidney diseases and, also, represents the major determinant of renal function decline [2]. A wealth of studies has indicated that renal function decline correlates well with the increasing risks for all-cause mortality, cardiovascular events and hospitalization [1,3,4]. Despite that current available therapies have targeted traditional risk factors for renal function decline—namely, hypertension, hyperglycemia and hyperlipidemia, nearly half of CKD patients still experience progressive renal function decline and eventual end-stage renal disease [5]. Hence, a novel treatment modality to tackle renal fibrosis and halt the progression of CKD is urgently needed.

Infiltrated renal macrophages play pivotal roles in the homeostasis of renal fibrogenesis following initial renal insult, either ischemic, immunologic, mechanical or toxic damage [6]. Macrophages are highly plastic and differentiate into different phenotypes in response to the local environments. Macrophage phenotypes can be broadly categorized into the proinflammatory “M1” macrophages (characterized by inducible nitric oxide synthase (iNOS)) and the anti-inflammatory, reparative “M2” macrophages (characterized by arginase-1 (Arg1)) [6]. M2 macrophages can be further classified into M2a and M2c subpopulations by the presence of C-type lectin domain family 7 member A (CLEC7A) and signaling lymphocytic activation molecule (SLAM), respectively [7]. These macrophage subsets play distinct roles in the wound-healing process following tissue injury [8]. During the normal wound-healing process, the initial proinflammatory milieu recruits M1 macrophages to induce apoptosis and eliminate the pathogen and necrotic tissue. Thereafter, anti-inflammatory M2 macrophages predominate in the later tissue repair stage to activate re-epithelialization and neoangiogenesis in the injured area. Finally, a resolution stage ends the whole healing process by promoting the apoptosis of recruited immune cells, suppression of inflammation and tissue remodeling [8]. Nonetheless, dysregulation of the M1-to-M2 transition in a normal wound-healing process would lead to pathologic fibrosis and tissue scarring [8]. Recent studies further suggest that the excessive activation of M1 macrophages and certain profibrotic M2 macrophages both contribute to the development of fibrosis formation [9].

M2 macrophages may function as a double-edged sword in regulating renal fibrosis. M2 macrophages help control inflammation through releasing interleukin (IL)-10, Arg1, transforming growth factor-β (TGF-β) and heme oxygenase-1 [9]. On the other hand, the chronic activation of M2 macrophages can activate resident fibroblasts through the release of TGF-β, platelet-derived growth factor, vascular endothelium growth factor, insulin-like growth factor-1 and galactin-3 [9]. In this regard, M2 macrophages are proposed to be profibrotic in the renal fibrosis model of unilateral ureteral obstruction (UUO), and depletion of these M2 macrophages in UUO should be beneficial [9,10]. However, not all macrophage depletion strategies result in a reduction in fibrosis in UUO [10]. Inhibition of the c-fms kinase almost suppresses all infiltrating macrophage numbers in day 14 obstructed kidneys but does not change the course of fibrosis, suggesting some antifibrotic M2 macrophages are also depleted [11]. Until now, the functional roles of the M2 macrophage subpopulation in renal fibrogenesis remained unclear and conflicting. M2a and M2c macrophages are initially found to be anti-inflammatory and reparative in murine Adriamycin nephrosis [12]. Nonetheless, two recent studies indicate that M2a macrophages are upregulated in endometrial fibrosis and skeletal muscle fibrosis [13,14], suggesting M2a macrophages may exhibit a profibrotic feature in chronic fibrotic diseases. Therefore, further elucidating the roles of the M2a and M2c subsets may help delineate the complex fibrogenic process in UUO.

Emerging evidence indicates that epigenetic modulation of the chromatin state is crucial in determining the progression of CKD and macrophage polarization [15,16]. The histone acetylation status has recently been found to associate with certain kidney diseases and renal fibrogenesis [17]. Histone deacetylases (HDACs) are a group of enzymes that exert epigenetic effects by altering the acetylation status of histone and nonhistone proteins [18]. Although potential favorable effects of HDAC inhibitors have been found in animal models of acute kidney injury [19], diabetic nephropathy [20] and Adriamycin nephropathy [21], the roles of HDAC inhibitors in UUO and macrophage plasticity remain incompletely understood. Marumo et al. found that the expression of HDAC1 and HDAC2 are upregulated in obstructed kidneys and contribute to proinflammatory and fibrotic responses [22]. Nonetheless, whether HDAC inhibition regulates the phenotypic change of renal interstitial macrophages in UUO is still unclear. Previously, HDAC3-deficient bone marrow-derived macrophages displayed an M2-polarized IL-4-induced alternatively activated phenotype [11], implying HDAC inhibition may contribute to M2 macrophage polarization. Currently, there is no study exploring the interaction between histone acetylation and macrophage subsets in kidney diseases. Hence, we aimed to investigate whether HDAC inhibition attenuates renal fibrosis through modulating the phenotype of renal interstitial macrophages.

In this study, we found the distinct expression of M2a and M2c subset macrophages in obstructed kidneys. An increased M2a macrophage infiltrate correlated with a higher extent of renal interstitial fibrosis. Interestingly, the administration of trichostatin A (TSA), an HDAC inhibitor, suppressed M2a macrophage infiltration, enhanced M2c macrophage expression and attenuated renal fibrosis in UUO. TSA also repressed the expression of proinflammatory and profibrotic molecules in cultured M2a macrophages and inhibited the activation of renal myofibroblasts. Our study is the first to demonstrate that TSA modulates the renal macrophage M2 subpopulation to inhibit renal fibrosis.

## 2. Results

### 2.1. Infiltration of Interstitial Macrophages Correlates with Fibrosis in Obstructed Kidneys

**Figure 1:**
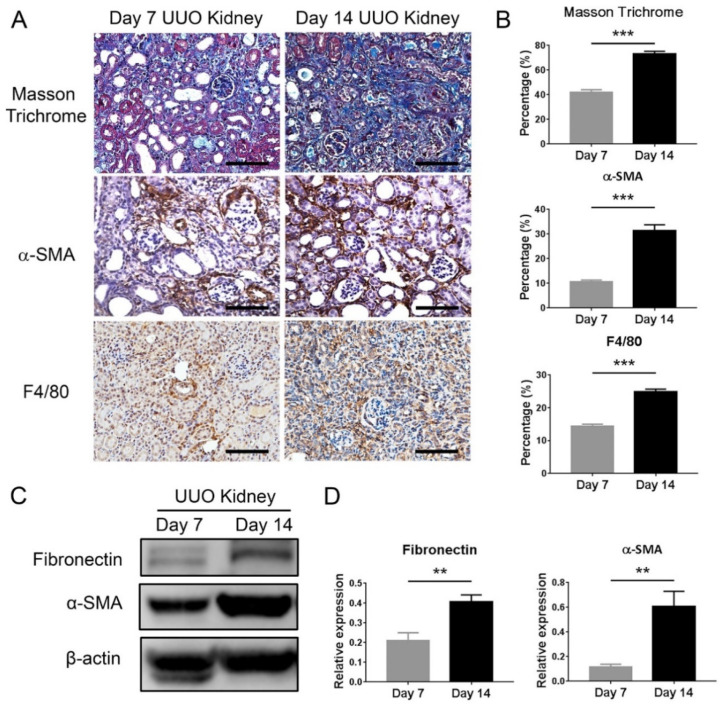
Macrophage infiltration correlates with renal interstitial fibrosis following a unilateral ureteral obstruction (UUO) injury. (**A**) Representative images of the Masson trichrome staining showed that the extent of fibrosis (blue staining) progressively increased in day 14 UUO kidneys as compared to day 7 ones. Immunohistochemistry demonstrated that more α-smooth muscle actin (α-SMA)-positive myofibroblasts and F4/80-positive macrophages accumulated in day 14 UUO kidneys. Scale bar = 100 μm. (**B**) Quantification of the fibrosis extent, α-SMA-positive and F4/80-positive areas. ** *p <* 0.01 and *** *p* < 0.001 by the unpaired Student’s *t*-test; *n* = 5 for each group. (**C**) Western blot analysis of fibronectin and α-SMA expression in day 7 and day 14 obstructed kidneys. β-actin served as the loading control. (**D**) Quantification of the Western blot analyses. ** *p <* 0.01 and *** *p <* 0.001 by the unpaired Student’s *t*-test; *n* = 5 for each group.

To investigate the role of macrophage infiltration in renal fibrosis, we compared five mice kidneys harvested seven days after UUO to five mice kidneys harvested 14 days after UUO. Masson trichrome staining indicated that the area of renal tubulointerstitial fibrosis increased with the time of UUO. Moreover, immunohistochemistry showed α-smooth muscle actin (α-SMA)-positive myofibroblasts progressively increased in the day 14 obstructed kidneys as compared to the day 7 ones. Notably, the area of F4/80 (pan-macrophage marker)-positive macrophage infiltrate also correspondingly increased in the day 14 obstructed kidneys (Figure 1), indicating that interstitial macrophages did play an essential role in the development of renal fibrosis.

### 2.2. Preferential Accumulation of M1 and M2a Macrophages in Obstructed Kidneys

**Figure 2:**
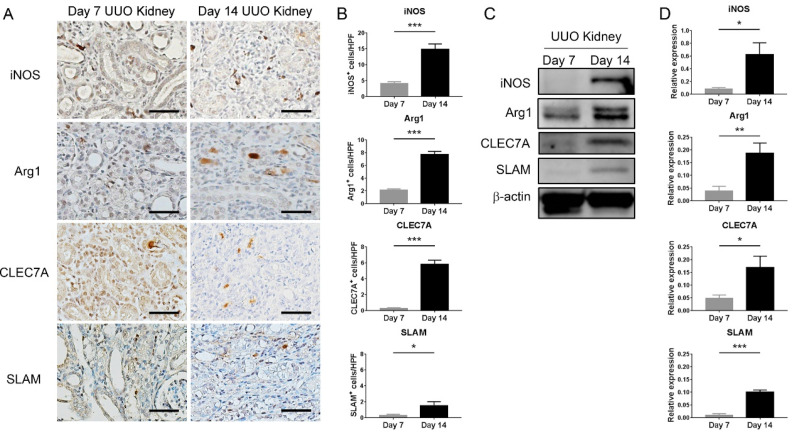
M1 and M2a macrophage infiltrates predominate in the late stage of unilateral ureteral obstruction (UUO). (**A**) Representative immunohistochemical photomicrographs of inducible nitric oxide synthase (iNOS), arginase-1 (Arg1), C-type lectin domain family 7 member A (CLEC7A) and signaling lymphocytic activation molecule (SLAM) in day 7 and day 14 obstructed kidneys. Scale bar = 25 μm. (**B**) Quantification of the iNOS-, Arg1-, CLEC7A- and SLAM-positive interstitial cells in day 7 and day 14 obstructed kidneys. * *p <* 0.05 and *** *p <* 0.001 by the unpaired Student’s *t*-test; *n* = 5 for each group. (**C**) Western blot analyses of iNOS, Arg1, CLEC7A and SLAM expressions in day 7 and day 14 obstructed kidneys. β-actin served as the loading control. (**D**) Quantification of the Western blot analyses. * *p <* 0.05, ** *p <* 0.01 and *** *p <* 0.001 by the unpaired Student’s *t*-test; *n* = 5 for each group.

As macrophage plasticity is critical for regulating renal fibrosis [8], next, we analyzed the phenotypes of macrophages at different time points of UUO. Immunohistochemistry showed that both iNOS-positive M1 macrophages and Arg1-positive M2 macrophages progressively accumulated in the day 14 obstructed kidneys. Thereafter, we explored the M2 macrophage subpopulation in UUO. Interestingly, CLEC7A-positive M2a macrophages predominantly expressed in the day 14 obstructed kidneys. By contrast, the number of SLAM-positive M2c macrophages was only slightly increased over time (Figure 2A,B). Western blot analyses also showed the consistent results that the expression of M1 and M2a markers progressively increased with the time of UUO (Figure 2C,D). These data suggest that M1 and M2a macrophages are involved in the development of renal fibrosis.

### 2.3. HDAC Inhibition Represses Renal Fibrosis and Macrophage Infiltration in UUO

**Figure 3:**
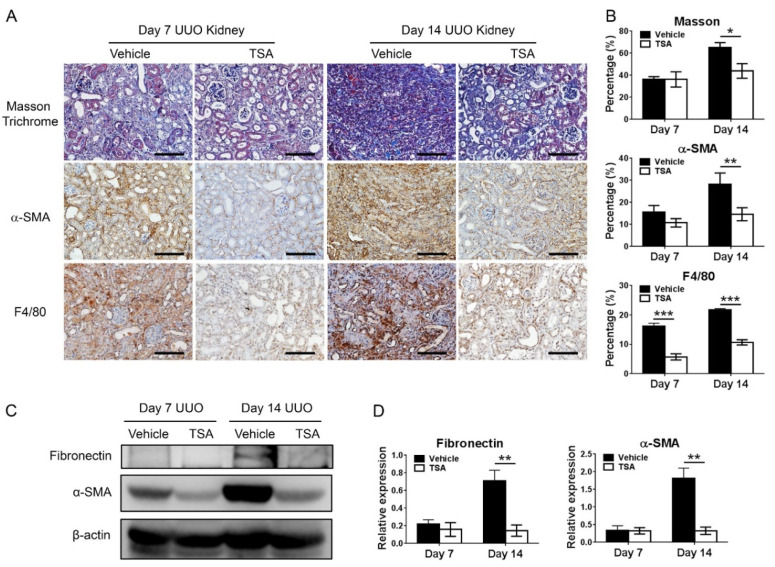
Trichostatin A (TSA) ameliorates renal inflammation and fibrosis in unilateral ureteral obstruction (UUO). (**A**) Representative images of Masson trichrome staining of obstructed kidneys at 7 days and 14 days following UUO in the vehicle and TSA groups. The TSA treatment markedly reduced the extent of interstitial fibrosis (blue area). Immunohistochemical staining for α-smooth muscle actin (α-SMA, myofibroblast marker) and F4/80 (pan-macrophage marker) in day 7 and day 14 obstructed kidneys. Scale bar = 100 μm. (**B**) Quantification of the fibrosis extent, α-SMA-positive and F4/80-positive areas. * *p <* 0.05, ** *p <* 0.01 and *** *p <* 0.001 by the unpaired Student’s *t*-test; *n* = 5 for each group. (**C**) Western blots of fibronectin and α-SMA in day 7 and day 14 UUO kidneys treated with the vehicle or TSA. β-actin served as the loading control. (**D**) Quantification of fibronectin and α-SMA expression levels. ** *p <* 0.01 by the unpaired Student’s *t*-test; *n* = 5 for each group.

Given that increased histone deacetylation may be associated with renal fibrogenesis [17], we then investigated the therapeutic effect of TSA in UUO. The administration of TSA significantly reduced the extent of α-SMA-positive myofibroblasts and the area of renal interstitial fibrosis in UUO. Moreover, TSA also decreased the extent of interstitial macrophage infiltrate (Figure 3A,B). Western blot analysis also found that TSA reduced the expression of the extracellular matrix (fibronectin) and α-SMA in obstructed kidneys (Figure 3C,D). 

### 2.4. HDAC Inhibition Promotes an M1-to-M2 Phenotypic Transition and Skews the M2 Macrophage Phenotype Towards an M2c Feature

**Figure 4:**
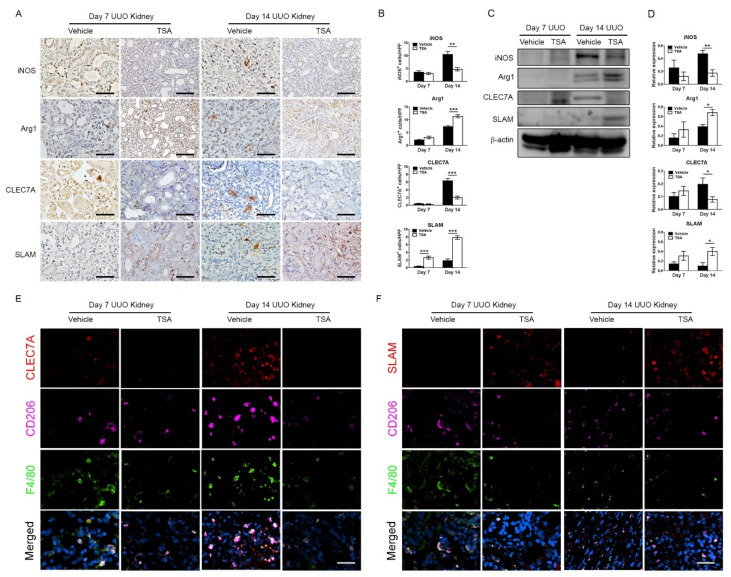
Trichostatin A (TSA) suppresses M1 and M2a macrophage infiltration but promotes M2c macrophage infiltration in unilateral ureteral obstruction (UUO). (**A**) Representative immunohistochemical images of inducible nitric oxide synthase (iNOS, M1 macrophage marker), arginase-1 (Arg1, pan-M2 macrophage marker), C-type lectin domain family 7 member A (CLEC7A, M2a macrophage marker) and signaling lymphocytic activation molecule (SLAM, M2c macrophage marker) in day 7 and day 14 obstructed kidneys either treated with the vehicle or TSA. Scale bar = 25 μm. (**B**) Quantification of the iNOS-, Arg1-, CLEC7A- and SLAM-positive interstitial cells in day 7 and day 14 obstructed kidneys treated with the vehicle or TSA. ** *p <* 0.01 and *** *p <* 0.001 by the unpaired Student’s *t*-test; *n* = 5 for each group. (**C**) Western blots of iNOS, Arg1, CLEC7A and SLAM in day 7 and day 14 obstructed kidneys either treated with the vehicle or TSA. β-actin served as the loading control. (**D**) Quantification of iNOS, Arg1, CLEC7A and SLAM expression levels in obstructed kidneys. * *p <* 0.05 and ** *p <* 0.01 by the unpaired Student’s *t*-test; *n* = 5 for each group. (**E**) Immunofluorescent staining of CLEC7A, CD206 and F4/80 in obstructed kidneys. Scale bar = 25 μm. (**F**) Immunofluorescent staining of SLAM, CD206 and F4/80 in obstructed kidneys. Scale bar = 25 μm. White color indicates colocalization in the merged panels.

To explore the role of HDAC inhibition on macrophage plasticity, we analyzed the phenotypes of interstitial macrophages in UUO after the administration of TSA. Upon treatment with TSA, immunohistochemistry showed that the infiltration of iNOS-positive M1 macrophages significantly decreased as compared to the vehicle group. Conversely, Arg1-positive macrophages were significantly upregulated in the TSA-treated kidneys following 14 days of obstruction (Figure 4A,B). Remarkably, TSA increased the number of SLAM-positive M2c macrophages but decreased the number of M2a macrophages. Immunofluorescent staining showed that both CLEC7A and SLAM colocalized with F4/80+CD206+ cells in obstructed kidneys, supporting that CLEC7A-positive and SLAM-positive cells represented M2a and M2c macrophages, respectively. Immunoblot confirmed that TSA downregulated the expression of iNOS and CLEC7A, as well as upregulated the expression of Arg1 and CLEC7A (Figure 4C–F). These data indicated that TSA suppressed the accumulation of proinflammatory M1 macrophages and profibrotic M2a macrophages in UUO. Instead, TSA increased the anti-inflammatory M2c macrophage infiltration, which limited the extent of inflammation and inhibited the activation of myofibroblasts and deposition of the extracellular matrix. 

### 2.5. HDAC Inhibition Suppresses Proinflammatory and Profibrotic Phenotypes in Cultured M2 Macrophages

**Figure 5:**
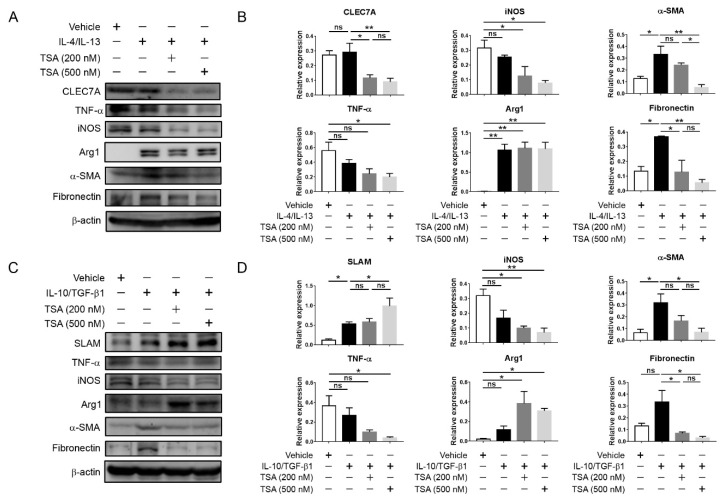
The effect of trichostatin A (TSA) on proinflammatory and profibrotic phenotypes of M2a and M2c macrophages. (**A**) Western blots of C-type lectin domain family 7 member A (CLEC7A), proinflammatory (tumor necrosis factor-α (TNF-α) and inducible nitric oxide synthase (iNOS)) and profibrotic (α-smooth muscle actin (α-SMA) and fibronectin) expressions in J774A.1 macrophages treated with the indicated conditions for 24 h. Concentrations of interleukin (IL)-4 and IL-13 were both 20 ng/mL. β-actin served as the loading control. (**B**) Quantification for the levels of indicated proteins. * *p <* 0.05 and ** *p <* 0.01 by ANOVA, followed by Tukey’s post hoc multiple comparison test; *n* = 3 for each group. (**C**) Western blots of SLAM, proinflammatory (TNF-α and iNOS) and profibrotic (α-SMA and fibronectin) expressions in J774A.1 macrophages treated with the indicated conditions for 24 h. Concentrations of IL-10 and transforming growth factor (TGF)-β1 were both 20 ng/mL. β-actin served as the loading control. (**D**) Quantification for the levels of indicated proteins. * *p <* 0.05 and ** *p <* 0.01 by ANOVA, followed by Tukey’s post hoc multiple comparison test; *n* = 3 for each group.

To further clarify the effect of HDAC inhibition on the proinflammatory and profibrotic phenotypes of macrophages, J774A.1 macrophages were stimulated with IL-4/IL-13 or IL-10/TGF-b1 in the presence or absence of TSA for 24 h. IL-4/IL-13 induced the expression of Arg1 and CLEC7A in J774A.1 macrophages and polarized the cells towards an M2a phenotype. M2a J774A.1 macrophages increased the expression of profibrotic α-SMA and fibronectin, which were suppressed by TSA treatment. Furthermore, the amounts of proinflammatory tumor necrosis factor-α and iNOS in the M2a macrophages were also downregulated by TSA (Figure 5A,B). In contrast, IL-10/TGF-β1 induced the SLAM expression in J774A.1 macrophages and skewed the cells towards an M2c phenotype. The TSA treatment enhanced the SLAM expression in M2c J774A.1 macrophages and also further suppressed the amounts of TNF-α, iNOS, α-SMA and fibronectin in a dose-dependent manner (Figure 5C,D).

### 2.6. HDAC Inhibition Attenuates TGF-β1-Activated Renal Tubular Epithelial Cells and Fibroblasts

**Figure 6:**
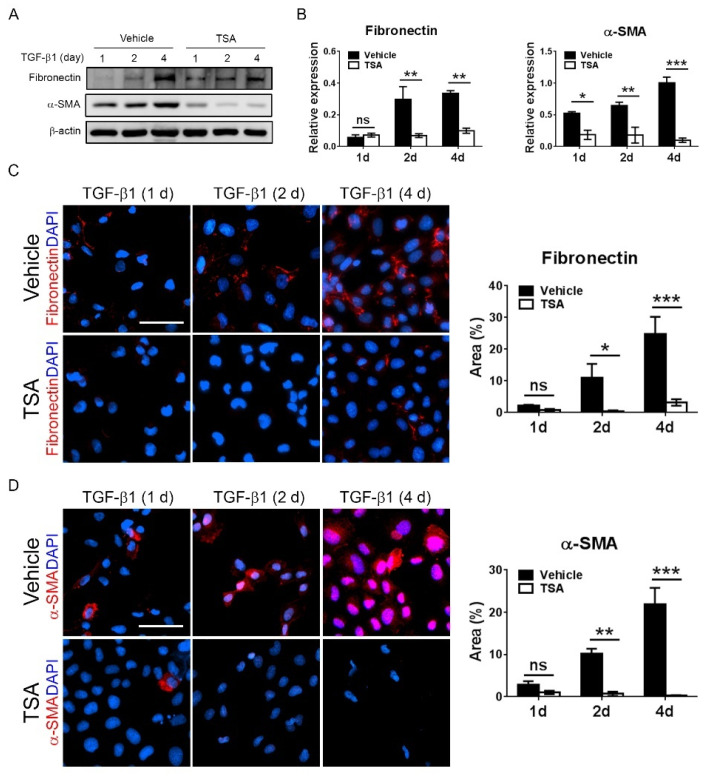
The effect of trichostatin A (TSA) on the activation of renal myofibroblasts. (**A**) Western blots of fibronectin and α-smooth muscle actin (a-SMA) expressions in transforming growth factor-β1 (TGF- β1, 20 ng/mL)-stimulated renal tubular epithelial NRK-52E cells at the indicated time. β-actin served as the loading control. (**B**) Quantification for the levels of indicated proteins. * *p <* 0.05, ** *p <* 0.01 and *** *p <* 0.001 by ANOVA, followed by Tukey’s post hoc multiple comparison test. ns, nonsignificance. *n* = 3 for each group. (**C,D**) Immunofluorescent staining of fibronectin and α-SMA in the TGF-β1-treated NRK-52E cells at the indicated time. Scale bar = 50 μm. * *p <* 0.05, ** *p <* 0.01 and *** *p <* 0.001 by ANOVA, followed by Tukey’s post hoc multiple comparison test. ns, nonsignificance. *n* = 3 for each group.

TGF-β1 is a well-known predominant mediator of the activation of renal myofibroblasts and resultant fibrogenesis in UUO [23]. To substantiate the role of HDAC inhibition on TGF-β1-induced renal fibrosis, renal tubular epithelial NRK-52E cells and renal NRK-49F fibroblasts were stimulated with TGF-β1 in the presence or absence of TSA. Western blotting revealed that TGF-β1 upregulated the expression of α-SMA and fibronectin in NRK-52E cells in a time-dependent manner. The treatment with TSA abrogated TGF-β1-induced fibrogenesis in NRK-52E cells (Figure 6A,B). These effects were further confirmed by immunofluorescent staining (Figure 6C,D). In NRK-49F fibroblasts, the TSA treatment also repressed TGF-β1-induced α-SMA expression (Supplementary Figure S1). These data indicate that TSA reduced renal fibrosis through attenuating the activation of myofibroblasts by TGF-β1. 

**Figure 7:**
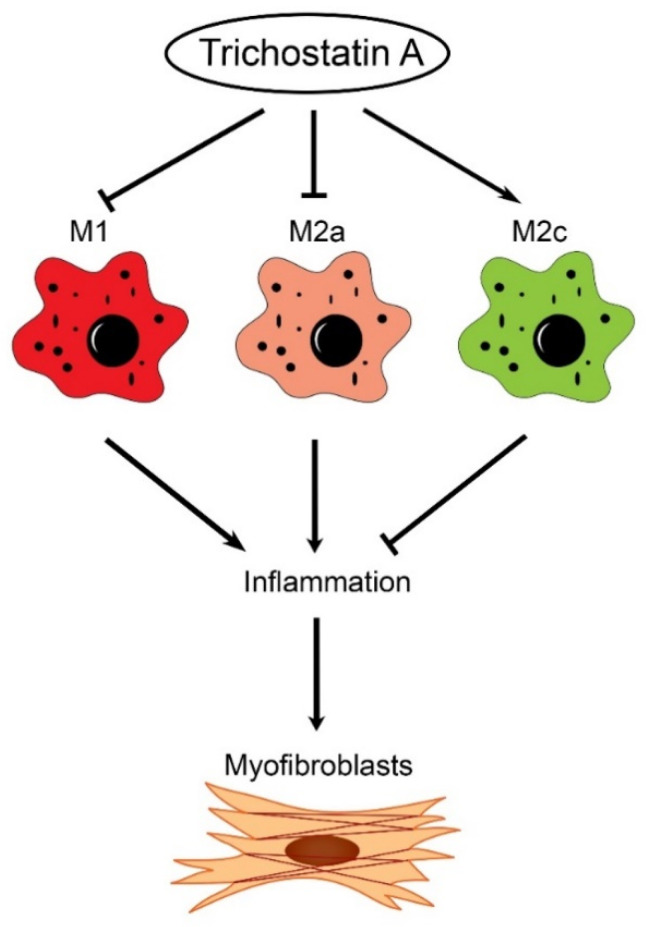
Proposed immunomodulatory and antifibrotic mechanism by trichostatin A in renal fibrosis. In a mice model of unilateral ureteral obstruction, the administration of trichostatin A suppresses the infiltration of proinflammatory M1a and M2a macrophages, as well as promotes the accumulation of anti-inflammatory M2c macrophages, thereby limiting excessive inflammation. Accordingly, the activation of myofibroblasts is limited, and renal fibrosis is attenuated.

Our findings are summarized schematically in Figure 7. In renal fibrogenesis, the accumulation of M1 and M2a macrophages enhances inflammation and activates myofibroblasts. Trichostatin A suppresses the infiltration of proinflammatory M1 and M2a macrophages and increases the infiltration of anti-inflammatory M2c macrophages, thereby inhibiting the activation of renal myofibroblasts and resultant renal fibrosis.

## 3. Discussion

This present study showed that HDAC inhibition by TSA reduced renal fibrosis through modulating the M2 macrophage subpopulation. We found that a TSA treatment significantly decreased the extent of interstitial fibrosis, myofibroblast accumulation and macrophage infiltration in the obstructed kidneys. Interestingly, TSA downregulated iNOS expression and upregulated Arg1 expression in the whole kidney protein expression levels. Consistently, immunochemistry also found that TSA suppressed the infiltration of iNOS-positive M1 macrophages and promoted the accumulation of Arg1-positive M2 macrophages in the obstructed renal interstitial areas. Specifically, TSA suppressed the accumulation of CLEC7A-positive M2a macrophages but increased the number of SLAM-positive M2c macrophages. These data indicated that the increment of M2 macrophages by TSA in UUO was preferentially attributed to the increased accumulation of M2c macrophages. Consistently, we also found that TSA suppressed CLEC7A expression in M2a macrophages and enhanced SLAM expression in M2c macrophages in vitro. Notably, TSA further downregulated the expression of proinflammatory and profibrotic factors in M2a macrophages. Moreover, TSA attenuated the TGF-β1-induced expression of α-SMA and fibronectin in cultured renal tubular epithelial cells and fibroblasts, indicating that TSA also attenuated the activation of renal myofibroblasts through inhibiting the epithelial-mesenchymal transition. Collectively, our findings indicated that TSA suppressed renal fibrogenesis through increasing the anti-inflammatory and antifibrotic M2 subpopulation. 

Conflicting results exist regarding the role of M2 macrophages on renal fibrosis in UUO [9], probably related to the heterogeneity of the M2 subpopulation. Previously, M2 macrophages were proposed to be profibrotic, because the macrophage infiltrate in the UUO-kidney has a predominant M2 phenotype and the percentage of M2 macrophages increases along with the duration of obstruction [10]. If this is the case, the depletion of all macrophages in the later stage of UUO should mitigate renal fibrosis. However, not all macrophage ablation strategies result in the improvement of renal fibrosis in UUO [10]. The depletion of infiltrating macrophages through targeting the c-fms kinase fails to attenuate the extent of renal fibrosis in day 14 obstructed kidneys [11], suggesting certain antifibrotic M2 macrophages are also depleted and also highlighting the complex functional heterogeneity of the M2 macrophage population [24]. Our study found that the TSA treatment resulted in fibrosis reduction, the downregulation of iNOS-positive M1 macrophages and CLEC7A-positive M2a macrophages and the upregulation of SLAM-positive M2c macrophages. In line with our observations, Wang et al. found that M2a macrophages are associated with the accumulation of collagen and muscle fibrosis [14]. Moreover, Duan et al. showed that the adoptive transfer of M2a macrophages significantly increased the extent of fibrosis in endometriosis [13], suggesting that M2a macrophages are profibrotic and critically involved in fibrogenesis. The controversy regarding the profibrotic or antifibrotic roles of M2 macrophages may be reconciled by the balance of distinct M2a and M2c macrophages in chronic fibrotic disorders.

Recent attention has focused on the significance of post-translational histone modifications in determining the progression of CKD and macrophage polarization [15,16]. Nonetheless, the role of epigenetic regulation on macrophage phenotypes in UUO remains elusive. The present study found that HDAC inhibition by TSA significantly alleviated renal inflammation and fibrosis. We also found that TSA inhibited the infiltration of M1 and M2a macrophages, enhanced the infiltration of M2c macrophages and directed an M1-to-M2c switch. The TSA-induced upregulation of M2c macrophages correlated with a reduction of renal interstitial fibrosis, and TSA also enhanced the SLAM expression and suppressed profibrotic and proinflammatory phenotypes in cultured M2c macrophages. These data supported that TSA repressed renal fibrosis through augmenting the expression of antifibrotic M2 macrophages. Although we found that TSA reduced UUO-induced renal fibrosis through modulation of the M2 macrophage subpopulation, our findings may not be extrapolated to renal fibrosis attributed to other insults, such as toxin, diabetes or autoimmune diseases. Clearly, more studies will be required to confirm the immunomodulatory and antifibrotic effects of TSA in other models of kidney diseases. In consistency with our findings, Pang et al. found that TSA can mitigate the extent of renal fibrosis in obstructive nephropathy, but the role of TSA on renal inflammation is undefined in their study [25]. A later study by Marumo et al. also found that TSA suppressed tubulointerstitial fibrosis in obstructed kidneys and further showed that TSA reduced renal inflammation in terms of the downregulation of EMR1 and MCP1 expressions, as well as decreased the number of infiltrated macrophages [22]. However, the effect of HDAC inhibition on the phenotypic transition of macrophages was not examined [22]. Taken together, our study provided novel evidence that TSA attenuated renal fibrosis through suppressing the profibrotic M2a macrophage and promoting anti-inflammatory, antifibrotic M2c macrophages. In conclusion, our study showed that HDAC inhibition by TSA significantly attenuated renal fibrosis through promoting an M1-to-M2c macrophage transition in obstructed kidneys. Our study not only added to the knowledge of antifibrotic M2 macrophages but, also, provided the bench evidence that pharmacological HDAC inhibitors can be applied to clinical treatments of renal fibrosis in the future.

## 4. Materials and Methods

### 4.1. Animals

Male 6-to-8-week-old C57BL/6J mice weighing 20-25 g were purchased from the National Laboratory Animal Center (Taipei, Taiwan) and housed at the Laboratory Animal Center of the National Yang-Ming University (Taipei, Taiwan). The mice were raised in a sound-attenuated, temperature-controlled (22 ± 1 °C) room with a 12-h light/dark cycle. Standard rodent chow and drinking water were supplied ad libitum. All experimental procedures conformed to the Guide for the Care and Use of Laboratory Animals published by the National Institutes of Health. The study was approved by the Institutional Animal Care and Use Committee of the National Yang-Ming University under the license number 1041244.

### 4.2. Cell Culture and Treatment

Mouse J774A.1 macrophages (Bioresource Collection and Research Center, Hsin-Chu, Taiwan) were cultured in Dulbecco’s modified Eagle’s medium (10-013-CM, Corning Inc., Corning, NY, USA) supplemented with 10% fetal bovine serum (Gibco, Grand Island, NY, USA) in a 5% CO_2_, 37 °C humidified incubator. To examine the effect of TSA on the macrophage subpopulation, J774A.1 macrophages were first seeded in 6-well dishes (5 × 10^6^ cells/well). Afterwards, J774A.1 macrophages were polarized into the M2a phenotype by stimulation with IL-4 (20 ng/mL, PeproTech, Rocky Hill, NJ, USA) and IL-13 (20 ng/mL, PeproTech) or into the M2c phenotype by stimulation with IL-10 (20 ng/mL, PeproTech) and TGF-b1 (20 ng/mL, PeproTech) for 24 h in the presence or absence of TSA (200 nM and 500 nM, T8552, Sigma-Aldrich, St. Louis, MO, USA) at different concentrations.

Normal rat renal tubular NRK-52E cells (Bioresource Collection and Research Center) were cultured in the low-glucose Dulbecco’s modified Eagle’s medium (10-014-CV, Corning Inc.) supplemented with 5% fetal bovine serum (Gibco). To determine the effect of TSA on renal myofibroblasts, NRK-52E were stimulated with TGF-β1 (20 ng/mL, PeproTech) for 1, 2 and 4 days in the presence or absence of TSA (500 nM, Sigma-Aldrich). 

### 4.3. Experimental UUO Model

After anesthesia, the UUO model was performed in mice by ligation of the left ureter with 5-O silk through a flank incision, as previously described [26]. After the surgery, the mice were recovered under a warming lamp. For HDAC inhibition experiments, TSA (1 mg/kg, Sigma-Aldrich) or the vehicle were injected intraperitoneally daily. The animals were euthanized, and the obstructed kidneys were harvested 7 and 14 days after UUO for further analyses.

### 4.4. Histological Analysis of the Kidneys

The kidney tissue was fixed with 4% phosphate-buffered formalin solution (Macron Chemicals, Center Valley, PA, USA), embedded in paraffin block and cut into 4-μm sections. For histological analysis, after deparaffinization and rehydration by xylene and graded alcohols, the sections were subjected to Masson trichrome staining according to the manufacturer’s instructions (Accustain, Sigma-Aldrich). Twenty randomly selected nonoverlapping high-power fields (40× objective) were evaluated for each mouse, and the average for each group was then analyzed. The fibrotic area was quantified by ImageJ software (1.52a, US National Institutes of Health, Bethesda, MD, USA).

### 4.5. Immunohistochemical Staining

Immunohistochemical staining was performed on formalin-fixed paraffin-embedded sections of obstructed kidneys, as previously described [27]. Briefly, after deparaffinization by xylene and rehydration by graded alcohols, consecutive 4-μm sections of kidneys were subjected to heat antigen retrieval in a microwave oven (650W, 12 min) in a 10-mM sodium citrate buffer (pH 6.0). Afterwards, endogenous peroxidase activity was quenched by 3% hydrogen peroxide (Sigma-Aldrich) for 10 min. Thereafter, tissue sections were incubated with the primary antibodies at 4 °C overnight and then with the secondary antibody (Envision^+^Dual Link System-HRP, Dako, Glostrup, Denmark) for 30 min at room temperature. Signals were developed with diaminobenzidine substrate-chromogen (DAB, Dako), which resulted in a brown-colored precipitate at the antigen site. Finally, sections were counterstained with a Gill’s hematoxylin (Merck, Darmstadt, Germany). Primary antibodies included F4/80 (1:100, Cat#sc-25830, Santa Cruz Biotechnology, Santa Cruz, CA, USA), iNOS (1:200, Cat#sc-651, Santa Cruz Biotechnology), Arg1 (1:200, Cat#sc-20150, Santa Cruz Biotechnology), α-SMA (1:200, Cat#ab5694, Abcam, Cambridge, UK), CLEC7A (1:50, Cat#TA322197, OriGene Technologies, Rockville, MD, USA) and SLAM (1:100, Cat#ab156288, Abcam). Twenty randomly selected nonoverlapping high-power fields (40× objective) at the renal cortex were evaluated for each mouse. Analysis of the DAB-positive area was carried out using Image J with the “Threshold Colour” plug-in (version 1.16, https://imagejdocu.tudor.lu/plugin/color/threshold_colour/start#threshold_colour).

### 4.6. Western Blotting

Western blotting analysis was performed as previously described [28]. Briefly, protein from the obstructed kidney tissue or cells was extracted in a radioimmunoprecipitation assay buffer containing a protease inhibitor cocktail (cOmplete-Mini, Roche, Indianapolis, IN, USA). The protein concentration was determined by a Bradford assay (Bio-Rad Laboratories, Montreal, Quebec, Canada), separated by sodium dodecyl sulfate-polyacrylamide gel electrophoresis and, subsequently, transferred to polyvinylidene fluoride membranes. The membranes were then probed with primary antibodies against fibronectin (1:1000, Cat#15613-1-AP, Proteintech Group, Chicago, IL, USA), α-SMA (1:5000, Cat#14395-1-AP, Proteintech Group), iNOS (1:1000, Cat#ab3523, Abcam), Arg1 (1:1000, Cat#93668S, Cell Signaling Technology, Boston, MA, USA), CLEC7A (1:500, Cat#TA322197, OriGene Technologies), SLAM (1:1000, Cat#ab156288, Abcam), TNF-α (1:1000, Cat#ab66579, Abcam) and β-actin (1:5000, Cat#60008, Proteintech Group) at 4 °C overnight. Afterwards, the membranes were incubated with horseradish peroxidase-conjugated secondary antibodies (Jackson ImmunoResearch, West Grove, PA, USA) at room temperature for 1.5 h, and the signals were developed using a West Femto Chemiluminescent Substrate kit (Thermo Fisher Scientific, Hudson, NH, USA). Bands were visualized and quantified using a ChemiDoc-It Imaging system (UVP, Cambridge, UK). Data were normalized to the β-actin expression.

### 4.7. Immunofluorescence

For in vivo experiments, paraffin-embedded 4-μm sections of obstructed kidneys were deparaffinized with xylene, rehydrated with graded alcohols and boiled in a 10-mM citrate buffer. Thereafter, the sections were blocked with hydrogen peroxide and then reacted with primary antibodies against F4/80 (1:100, Cat#MCA497R, Abd Serotec, Oxford, UK), CD206 (1:100, Cat#60143-1-Ig, Proteintech Group), CLEC7A (1:50, Cat#MBS9414183, MyBiosource, San Diego, CA, USA) or SLAM (1:100, Cat#ab156288, Abcam) at 4 °C overnight. Fluorescein isothiocyanate-conjugated goat anti-rat immunoglobulin G (IgG, 1:250, Cat#112-095-003, Jackson ImmunoResearch) and Alexa Fluor 647-conjugated donkey anti-mouse IgG (1:250, Cat# 715-605-151, Jackson ImmunoResearch) were used to visualize the location of F4/80 and CD206, respectively. Alexa Fluor 568-conjugated goat anti-rabbit IgG (1:250, Cat#A11011, Thermo Fisher Scientific) was used to visualize the location of CLEC7A and SLAM. Slides were then mounted with Fluoroshield Mounting Medium with DAPI (ab104139, Abcam).

For in vitro experiments, TGF-β1-stimulated NRK-52E cells were plated in chamber slides (μ-Slide 8-Well, Ibidi, Munich, Germany) for 1, 2 and 4 days in the presence or absence of trichostatin. Afterwards, the chamber slides were fixed with 4% paraformaldehyde for 10 min and blocked with 1% bovine serum albumin for 30 min. Thereafter, slides were incubated with primary antibodies against α-SMA (1:200, ab5694, Abcam) or fibronectin (1:200, 15613-1-AP, Proteintech) at 4 °C overnight, reacted with Alexa Fluor 568-conjugated goat anti-rabbit secondary antibody (1:200, Cat#A11011, Thermo Fisher Scientific) and then counterstained with DAPI (ab104139, Abcam).

### 4.8. Statistical Analysis

All values are expressed as mean ± SEM. Between-group comparisons were determined by the unpaired Student’s *t*-tests or ANOVA, followed by Tukey’s post hoc multiple comparison test. Statistical analysis was performed using the Statistical Analysis System (SAS, Version 9.4, SAS Institute, Cary, NC, USA). A value of two-sided *p* < 0.05 was considered statistically significant.

## Supplementary Materials

The following are available online at https://www.mdpi.com/1422-0067/21/17/5966/s1, Figure S1. The effect of trichostatin A (TSA) on the activation of renal fibroblasts.
