# Supplementary material for: Trichostatin A Alleviates Renal Interstitial Fibrosis Through Modulation of the M2 Macrophage Subpopulation
Source: Int J Mol Sci. 2020 Aug 19;21(17):5966. doi: 10.3390/ijms21175966 (PMC7503910; doi:10.3390/ijms21175966)
Supplement: Supplementary file 1 [file ijms-21-05966-s001.pdf]

Supplemental Information

Trichostatin A alleviates renal interstitial fibrosis through modulation of M2 macrophage subpopulation

Supplemental materials and methods

Cell culture and treatment

Normal rat renal tubular NRK-49F cells (Bioresource Collection and Research Center) were cultured in the low-glucose Dulbecco's Modified Eagle Medium (10-014-CV, Corning Inc.) supplemented with 5% fetal bovine serum (Gibco). To determine the effect of trichostatin A on the activation of renal fibroblasts, NRK-49F cells were stimulated with transforming growth factor-β1 (5 ng/mL, PeproTech) for 1 and 2 days in the presence or absence of trichostatin A (500 nM, Sigma-Aldrich).

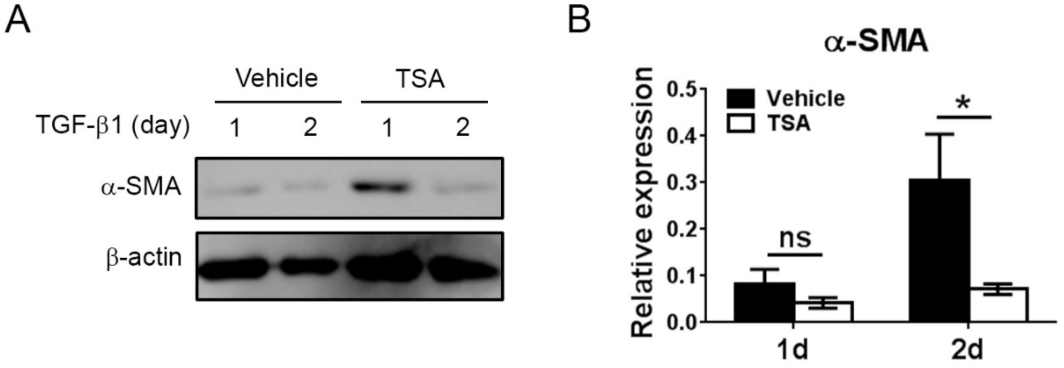

Supplemental Figure 1. The effect of trichostatin A (TSA) on the activation of renal fibroblasts. (A) Western blots of α-smooth muscle actin (α-SMA) expression in transforming growth factor-β1 (TGF-β1, 5 ng/mL)-stimulated renal NRK-49F fibroblasts at the indicated time. β-actin served as the loading control. (B) Quantification of the α-SMA expression, * $p < 0.05$ by the unpaired Student's t test. ns, non-significance. n = 3 for each group.
